# A Method for Investigating Population Declines of Migratory Birds Using Stable Isotopes: Origins of Harvested Lesser Scaup in North America

**DOI:** 10.1371/journal.pone.0007915

**Published:** 2009-11-25

**Authors:** Keith A. Hobson, Michael B. Wunder, Steven L. Van Wilgenburg, Robert G. Clark, Leonard I. Wassenaar

**Affiliations:** 1 Environment Canada, Saskatoon, Saskatchewan, Canada; 2 Department of Integrative Biology, University of Colorado Denver, Denver, Colorado, United States of America; University of Bristol, United Kingdom

## Abstract

**Background:**

Elucidating geographic locations from where migratory birds are recruited into adult breeding populations is a fundamental but largely elusive goal in conservation biology. This is especially true for species that breed in remote northern areas where field-based demographic assessments are logistically challenging.

**Methodology/Findings:**

Here we used hydrogen isotopes (δD) to determine natal origins of migrating hatch-year lesser scaup (*Aythya affinis*) harvested by hunters in the United States from all North American flyways during the hunting seasons of 1999–2000 (n = 412) and 2000–2001 (n = 455). We combined geospatial, observational, and analytical data sources, including known scaup breeding range, δD values of feathers from juveniles at natal sites, models of δD for growing-season precipitation, and scaup band-recovery data to generate probabilistic natal origin landscapes for individual scaup. We then used Monte Carlo integration to model assignment uncertainty from among individual δD variance estimates from birds of known molt origin and also from band-return data summarized at the flyway level. We compared the distribution of scaup natal origin with the distribution of breeding population counts obtained from systematic long-term surveys.

**Conclusions/Significance:**

Our analysis revealed that the proportion of young scaup produced in the northern (above 60°N) versus the southern boreal and Prairie-Parkland region was inversely related to the proportions of breeding adults using these regions, suggesting that despite having a higher relative abundance of breeding adults, the northern boreal region was less productive for scaup recruitment into the harvest than more southern biomes. Our approach for evaluating population declines of migratory birds (particularly game birds) synthesizes all available distributional data and exploits the advantages of intrinsic isotopic markers that link individuals to geography.

## Introduction

Juvenile production and mortality rates are important factors determining the population dynamics of many species because they directly limit the rate of recruitment into breeding populations. For migratory birds, juvenile mortality may be particularly pronounced during the initial southward movement from the northern natal site or during the first winter when young birds are most inexperienced [1]–[4]. However, identifying key geographical areas of avian productivity and mortality and quantifying the resulting impact on a species' population are difficult to measure directly. For species with geographically broad breeding and/or wintering distributions, it is often unclear whether productivity and mortality factors vary spatially across distributed or regional populations. This problem has led to immense interest in finding new ways of establishing and quantifying migratory connectivity between breeding and wintering populations, delineating migratory routes, and determining where the most mortality occurs [1], [5]. Such basic life-cycle connectivity information is especially crucial for migratory and game species whose populations may be declining but where the causes of mortality, both spatially and temporally, are unknown. The efficacy of extrinsic markers such as leg bands and satellite transmitters to answer questions related to continent-scale patterns of demography in most migratory populations is compromised by a combination of logistical and technological constraints associated with broad-scale marking programs; we describe herein an alternate approach to address these questions [6].

The lesser scaup (*Aythya affinis*) is a migratory diving duck and popular gamebird that breeds in the Prairie Pothole Region from the U.S. Midwest northward through much the boreal forest of Canada and Alaska [7]. This species winters broadly across the southern U.S., Cuba, and northern Mexico. The North American continental population of lesser scaup declined during the 1980s, and remains well below established conservation goals; however, the cause(s) of that population decline are not clear. Decreases in the ratio of juvenile∶adult lesser scaup in the fall game harvest from 1961 to 1996 suggest that overall population declines may be related to lower recruitment of juvenile scaup into the fall population [8], [9]. Reduced recruitment may arise from poor female body condition [10], [11], increased exposure to contaminants due to shifts in dependence on zebra mussels (*Dreissena polymopha*; [12], [13]) in the Great Lakes region, or more widespread effects of habitat quality changes in the boreal ecozone that may also be responsible for declines in other species [8]. Increased adult female mortality and hunting impacts have also been proposed to explain population declines, which appear to be more pronounced in the northern boreal forest of western Canada [9].

Stable hydrogen isotopes (δD) allow the tracking of migratory birds without the need for extrinsic markers [14], [15], and are particularly useful since the δD abundance patterns in foodwebs generally mimic well-known and predictable geospatial patterns in the hydrogen isotopic composition of precipitation across continents [16]. Because of this, δD values for juvenal feathers of precocial birds will in general reflect those of the food at their natal site, which are in turn related in large part to patterns of δD in precipitation. This phenomenon provides a means to elucidate migratory connectivity through feather tissue assays, and thereby to determine the relative importance of various breeding areas for the recruitment of juveniles into fall migratory or wintering populations. Recently, geographical origins of juvenile and adult sandhill cranes (*Grus canadensis*) harvested during fall migration through the Central Flyway of North America were identified [17]. Production of prairie-nesting northern pintails (*Anas acuta*) was lower than expected by breeding population distribution based on isotope values in feather samples obtained from juvenile birds harvested in areas of western Canada [18]. To date, the isotope approach has not yet been applied to examine hypotheses related to demography and population trends in a declining species across the entire breeding and wintering range. In part this is due to challenges associated with characterizing isotopic variances in known-origin tissues (feathers, claws, etc.) across such wide ranges of geography [19].

Our objective here was to demonstrate an alternate and complementary approach to conventional mark-recovery methods for establishing migratory connectivity and relative productivity of a broadly distributed harvested species, and thereby to help better refine hypotheses related to species' population decline. We also tested whether juvenile recruitment of northern-breeding scaup was lower than that of southern birds. To this end, we utilized δD in feathers from hatch-year (HY) lesser scaup harvested during their fall migration throughout the Atlantic, Mississippi, Central, and Pacific Flyways in the United States. We used δD data from precipitation and from known-origin feathers, along with band-recovery information to model the relative importance of natal sites to scaup recruitment into migratory aggregations during fall. We compared how modeled geographic distribution of scaup origin matched that expected from population abundance determined from aerial breeding-pair surveys throughout the core breeding range.

## Methods

### Ethics Statement

This study was conducted under authority of the Canadian Council for Animal Care as reviewed by the University of Saskatchewan. All federal permits for the use of feather material were issued by the Canadian Wildlife Service and the U.S. Fish and Wildlife Service.

### Sample

We used previously published data [20] from known-origin feathers from pre-fledged juvenile scaup at seven different breeding locales (Table 1). For the fall migration and winters of 1999–2000 and 2000–2001 we obtained samples of primary feathers from 867 hunter-killed HY lesser scaup from wings contributed to the annual U.S. Fish and Wildlife Service (USFWS) harvest survey. These represented all available wings from all of the recognized flyways and the entire length of scaup hunting seasons. The outer primaries (P8 or occasionally P9) were subsampled from wings, placed in labeled envelopes and stored dry until laboratory analysis.

**Table 1:** Average and standard deviation of measured δD from feathers from hatch year Lesser scaup collected at seven sites across the breeding range, and associated predicted δD value for precipitation at each site (predicted from the model of [16]).

Site	Feather average δD (‰)	Feather SD	Predicted δD in Precipitation (‰)
1	−119.2	8.8	−90
2	−123.4	5.6	−105
3	−124.3	14.7	−117
4	−157.1	7.0	−127
5	−155.1	16.5	−132
6	−162.3	7.2	−138
7	−149.3	7.4	−139

### Stable Isotope Analyses

Primaries were cleaned of surface oils in 2∶1 chloroform∶methanol solvent rinses and prepared for δD analysis at the Stable Isotope Laboratory of Environment Canada, Saskatoon, Canada. Stable-hydrogen isotope analyses of feathers were conducted using the comparative equilibration method described by [21] through the use of previously calibrated keratin hydrogen-isotope reference materials. Isotopic measurements were performed on H_2_ derived from high-temperature flash pyrolysis of 350±10 ug feather subsamples and keratin standards using continuous-flow isotope-ratio mass spectrometry. Measurement of the three keratin laboratory reference materials (CFS, CHS, BWB) (corrected for linear instrumental drift) were both accurate and precise with typical mean δD±SD values of −147.4±0.8‰ (*n* = 5), −187±0. 6 ‰ (*n* = 5) and −108±0.3 ‰ (*n* = 5), respectively. A control keratin reference yielded a 6-month running SD of ±3.3 ‰ (*n* = 76). Replicate analysis of a single juvenile scaup feather, representing expected intra-sample isotopic heterogeneity, yielded a SD of ±1.2‰ (*n* = 5). All results are reported for non-exchangeable H expressed in the typical delta notation, in units of per mil (‰), and normalized on the Vienna Standard Mean Ocean Water – Standard Light Antarctic Precipitation (VSMOW-SLAP) standard scale.

### Construction of Spatially Explicit Probability Densities to Describe Potential Scaup Natal Origins

We created a probabilistic surface of potential natal origins for our sample of hunter-killed birds by combining (1) data from feathers of pre-fledged scaup as described above with (2) a model of expected δD values (an isoscape) formed primarily from data available from the Global Network of Isotopes in Precipitation (GNIP), and with (3) offsite band return data for lesser scaup and by (4) restricting the geographic range of potential sites to the known breeding range for scaup. We combined these information sources in a Bayesian framework that allowed us to model probability densities to describe potential natal origins for each of the 867 individual harvested HY scaup [c.f. 22], [23]. We then combined these geographically indexed densities into a single surface to describe the potential origins of all harvested birds combined.

Our model framework is rooted in the relationship between δD values in feathers (δD_f_) and modeled δD values in precipitation (δD_p_), which is generally non-random for most species of birds [15]. The modeled precipitation values are primarily output from a meteoric-pattern-corrected spatial interpolation of 40+-year average values for δD_p_ measured at the GNIP collection sites [24]. We used the output from this model at each geographic grid point to represent the expected δD_p_ at each grid point. Grid points were spaced at 0.33 degrees as determined by the spatial resolution in [16], [24].

**Figure 1:**
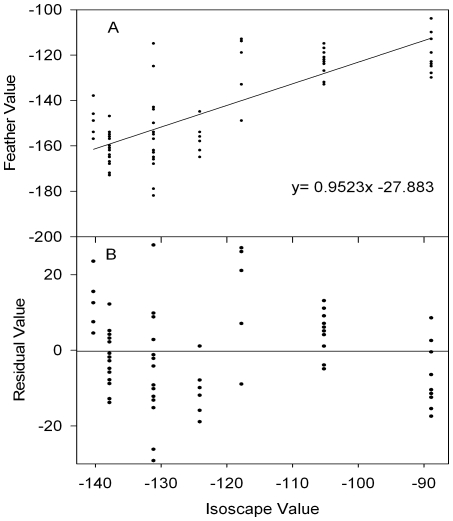
Functional relationship between expected δD_p_ for precipitation from the isoscape model of [16] and the observed feather δD_f_ values for scaup of known origin. Panel B shows the distribution of residuals from the regression in panel A.

To characterize the mean expected geographic matrix of δD_f_ values for HY scaup, we regressed δD_f_ values from feathers in pre-fledged scaup on the expected δD_p_ values at the sites where the feathers were sampled (Fig. 1). Approximately 64% of the variance in δD_f_ values was explained by this linear regression on the modeled values for growing season δD_p_ at the feather collection locations (δD_f_ = 0.9523 δD_p_ – 27.8‰), and the slope parameter was significantly different from zero (*p* = 2×10^−16^). We used this regression model as way to calibrate the precipitation isoscape to what we would expect for HY feathers, effectively modeling the mean expected feather isoscape for HY scaup [22].

The 95% confidence interval around the slope parameter of the regression was [0.7845, 1.1201] implying that the rescaling was likely to vary by approximately 24%. Similarly, the 95% confidence interval for the intercept was [−48.6, −7.0‰]. Given the degree of uncertainty in the regression parameters, it was reasonable to assume that the expected δD_f_ at any geographic coordinate was better characterized as a distribution of potential values rather than as the single value given by the calibrated δD_f_ isoscape. Therefore, we used the observed variance estimates for each location where pre-fledged scaup were sampled to model the uncertainty in δD_f_ when assigning an individual bird to a potential site of origin. The δD_f_ values for HY scaup at known locations varied within each collection site; sample standard deviations for sites ranged from 5.6‰ to 16.5‰ (Table 1). To model this variance in the observed variances across sites, we fit a gamma distribution to the series of within-site δD_f_ variance estimates given in Table 1, resulting in shape (*α* = 6.9087) and scale (*β* = 1.3910) parameters describing the distribution of *σ^2^*. We used a gamma distribution because variances are always non-negative and gamma distributions have support only over the non-negative domain. We used the fitted gamma distribution to scale the probability density for δD_f_ around the predicted values in the calibrated δD_f_ isoscape (see 22, 23).

We used Bayes' rule to compute the probability that location *x_i_* was the origin of the harvested individual given the observed feather value *y_jk_* where *j* indexes the flyway in which the bird was shot and *k* indexes the observation number within the flyway; so, *i* indexes the assignment locations and *j* indexes the collection locations. Our formulation of this probability was then
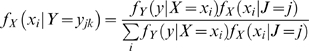
(0.1)where *f_X_*|*Y* is the spatially explicit posterior probability density function for *x_i_* as the location of origin, given the feather *y_jk_*. The random variable Y is continuous and represents the δD_f_ values for harvested HY scaup. The random variable X represents the cells within the isoscape, and in this instance has a dimension of 21,917 (will differ with the isoscape used and size of the species' range). The random variable J is categorical with dimension four, describing each of the four flyways from which harvested scaup were taken. To simplify the computation, *f_Y_* was modeled hierarchically using a Normal probability density with parameters *μ* (set equal to the predicted values for the *x_i_* from the rescaled δD_f_ isoscape) and *σ^2^∼Γ(α,β)*, with parameters *α* and *β* as described in the previous paragraph. Here, *f_Y_* can be interpreted as the probability of observing a sampled δD_f_ value at location *x_i_*, given the expected δD_f_ value for location *x_i_* from the calibrated isoscape and the among-individual variance associated with a typical sampled location.

Since we used band recovery frequency data from hunter-killed birds to parameterize the prior on X, the model for the prior on *f_X_* depended on where the bird was shot, as noted by the variable *J*. We structured the prior for *f_X_* differently for birds shot in each of the flyways. The index *j* could assume one of four values, one for each of the Pacific, Central, Mississippi, or the Atlantic flyways. The prior distributions for the flyways from which samples were taken are given in Table 2. For example, of the banded birds that were shot in the Atlantic flyway, 70% were banded in the Central flyway, 3% were banded in the Mississippi flyway and the remaining 27% were banded in the Pacific flyway. Consequently, if *x_i_* is a location within the breeding range for the Pacific flyway, and the feather value *y_jk_* was obtained from a bird collected in the Atlantic flyway, the prior weight on *x_i_* would be 0.27. If *x_i_* is a location within the Central flyway, the prior weight on *x_i_* would be 0.70 and it would be 0.03 if *x_i_* were within the Mississippi flyway.

**Table 2:** Prior probabilities (determined from hunter recoveries of banded birds) of a lesser scaup being harvested in a flyway.

		Shot in Atlantic	Shot in Mississippi	Shot in Central	Shot in Pacific
Origin					
	Central	0.70 (74)	0.75 (647)	0.86 (869)	0.29 (178)
	Mississippi	0.03 (3)	0.03 (4)	0.01 (35)	0.00 (1)
	Pacific	0.27 (28)	0.22 (97)	0.13 (251)	0.71 (441)

Numbers in parentheses are sample sizes. The Atlantic region does not appear as an origin because no scaup banded in the Atlantic flyway were recovered in our harvest sample here.

### Evaluation of Population Patterns

Since our goal was to explore the relationship between scaup productivity and abundance of various regions across the breeding range, we sought a geographically defined estimate of the number of young scaup produced across the breeding range. From each of the spatially-explicit probability densities for individual HY scaup, we sought a spatially-explicit binary response to define the likely area of origin for each individual. To accomplish this we determined the odds that any given assigned origin was correct relative to the odds that it was incorrect. We arbitrarily decided the geographic range corresponding to 2∶1 odds that a given scaup had originated from within that range would be reasonable for our purposes. Based on this decision, we identified the set of geographic coordinates defining the upper 33% of the cumulative density function and coded those as 1, with all others as 0. We then summed results for all individual scaup. These summations were qualitatively compared to the location-specific frequency distributions with estimated abundances of breeding adults for the various geographic locations within the breeding range.

Expected distributions of natal origins were inferred from year-specific (1999 and 2000) estimates of scaup (lesser and greater (*A. marila)* scaup combined) pair counts from the May Waterfowl Breeding Population and Habitat Survey [25]; tundra strata are dominated by greater scaup, and so these were excluded. Since the observational surveys do not cover the entire scaup range and isotopic contours intersect individual strata, we created interpolated surfaces from which to infer expected origins of birds. Accurate interpolation of scaup abundance across its breeding range involved modeling spatial structure within the data at two scales. Scaup abundance tends be lower in the southeast part of its range and higher to the northwest; furthermore, local scale (neighborhood) changes in abundance form an autocorrelated random component, where abundance at a site is correlated with abundance at neighboring sites. Due to the broad-scale spatial trend in scaup abundance, the assumption of a constant mean was violated for interpolations based on kriging. Therefore, global spatial trends in abundance were first modeled using trend surface analysis, where abundance was modeled using polynomials for latitude and longitude [26]. Local-scale autocorrelation in the residuals of the trend surface was then accounted for by Ordinary Point Cokriging. Kriging is a method of interpolating data values for unsampled areas based on underlying pairwise spatial correlations between neighbouring data points. This form of interpolation is accomplished by fitting empirical models (known as semivariograms) to describe increasing variance between pairs of data points as a function of the distance separating them. Kriging can be extended using co-kriging, where spatial covariance with another sampled variable may be used to improve the interpolation. In the case of scaup, which are generally philopatric, pair counts in year t+1 should be partially dependent on pair counts in year t. Therefore, co-kriging was conducted using scaup counts in the previous year as covariates (e.g., 1998 counts were covariates in analysis of 1999 data). Prior to all analyses, survey data were transformed (log(x+1)) to meet assumptions of homogeneity of variance. Semi-variogram models of local scale autocorrelation for kriging analysis were selected based on leave-one-out cross-validation. For all modeled years of survey data, rational quadratic models provided the best fit. Rational quadratic models describe increasing variance (semivariance, γ) between pairs of points with increasing distances separating them, given by the equation γ (h) = nugget + sill [(19(h/r)^2^)/(1+19(h/r)^2^)], where nugget is analogous to an intercept term describing sampling error or spatial variance at resolutions finer than the sampling scheme can detect, the sill is the semivariance at the distance where autocorrelation is no longer present (range), h is the lag (separation) distance between points, and r is the range (maximum distance over which the values are autocorrelated). All interpolations were conducted using Spatial Analyst™ (ESRI, Redlands, CA).

The model-estimated parameters for semivariograms are reported in Table 3. Interpolated surfaces were created from these models, with the trends in abundance due to latitude and longitude (trend surface) re-introduced and estimates back-transformed from the logarithm scale. We limited estimates for the Pacific population to an interpolation of scaup abundance in Alaska and the Yukon Territory alone, as most of the population likely originates from northern latitudes west of the continental divide.

**Table 3:** Parameter estimates for rational quadratic model semivariograms used for spatial interpolation of scaup pair counts in 1999 and 2000, from the May Waterfowl Breeding Population and Habitat Survey [27].

	Year	Covariate^b^	Trend removal	Lag (km)	Nugget	Range (km)	Partial Sill
Response^a^							
	1999	1998	2nd order	27.5	0.0559	87.5	0.1510
	2000	1999	1st order	25.0	0.0002	87.8	0.2115
Covariable^a^							
	1999	1998	1st order	27.5	0.0450	87.5	0.1486
	2000	1999	2nd order	25.0	0.0002	87.8	0.2086

a segment pair count in the year given.

b the year given is the year for which segment pair counts were used as covariates in interpolating the response variable.

All interpolations were conducted using Ordinary Point Cokriging of log(x+1) transformed data, detrended for global trends using polynomial regression, and using counts in the year previous to the data being interpolated as a covariates (see Methods).

In order to test if the assigned natal origins were correlated with scaup pair counts, we queried both the assigned origins surfaces (1999 and 2000) and interpolated abundance estimates with a point layer representing the cell centroids of the assigned origins surface. If natal origins are related to local scaup breeding abundance (pair counts), we would predict a statistically significant positive correlation between local breeding abundance and assigned natal origins for 1999 and 2000 respectively. We tested this hypothesis using Spearman's Rank-Order Correlation (rho) since data were not normally distributed.

## Results

**Figure 2:**
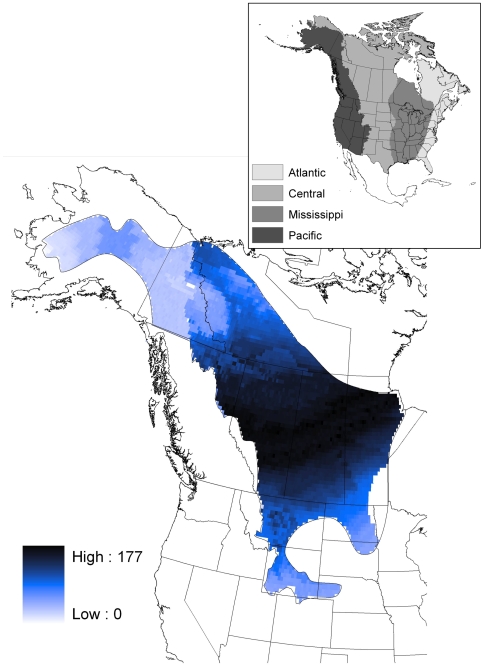
Geographic distribution of assigned sites of origin for hatching year scaup harvested in all flyways in 1999 and 2000 (n = 867) inferred from stable isotope (δD_f_) analysis of feathers. Inset depicts the flyway boundaries. Assignments were based on a Bayesian framework incorporating a spatially explicit isotope-based likelihood function with spatially explicit prior probabilities of being shot in a given flyway (based on banding records). The posterior probability density for each individual was then converted to a binary surface by assigning a “1” to all grid points with odds greater than 2∶1 relative to the highest density value and a “0” to all others. Each grid point shown here represents the sum of these individual-level binary grids, summed over all individuals across all four flyways and both years. Thus, a value of 177 represents a cell being consistent with representing an origin for 177 birds within the sample for example.

**Figure 3:**
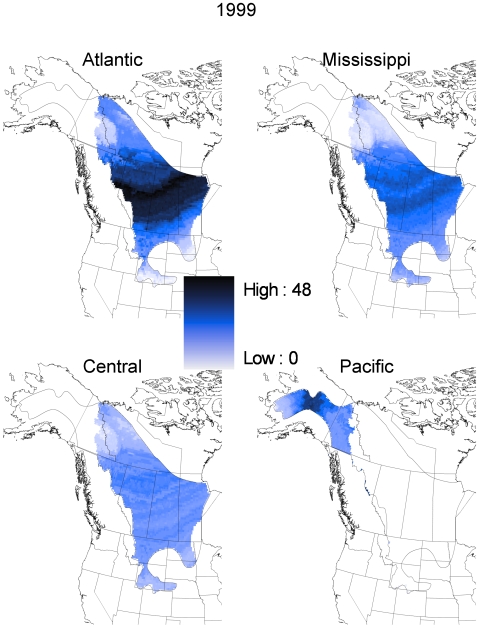
Geographic distribution of assigned sites of origin by flyway for hatching-year scaup harvested in 1999 inferred from stable isotope (δD_f_) analysis of feathers. Samples (Atlantic [n = 119], Mississippi [n = 99], Central [n = 85], Pacific [n = 109]) were assigned to a GIS-based model [16] of stable isotopes in precipitation (δD_p_) and recalibrating the surface to reflect δD in feathers via regression (see Methods). Assignments were based on a Bayesian framework incorporating a spatially explicit isotope-based likelihood function with spatially explicit prior probabilities of being shot in a given flyway based on banding records (see Methods and Table 2). The posterior probability density for each individual was then converted to a binary surface by assigning a “1” to all grid points with odds greater than 2∶1 relative to the highest density value and a “0” to all others. Each grid point shown here represents the sum of these individual-level binary grids, summed over all individuals.

**Figure 4:**
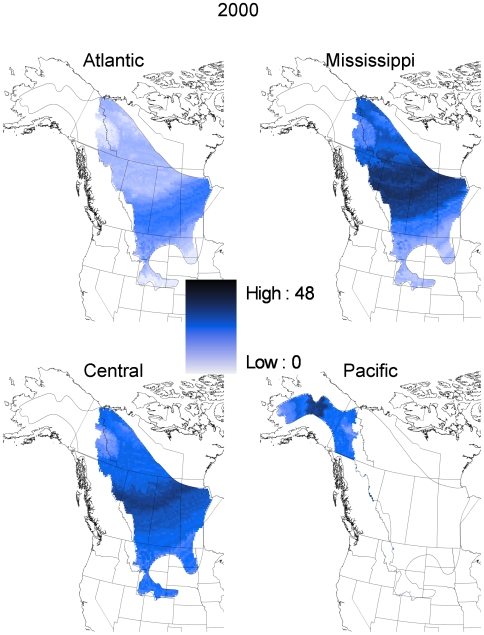
Geographic distribution of assigned sites of origin by flyway for hatching year scaup harvested in 2000 inferred from stable isotope (δD_f_) analysis of feathers. Samples (Atlantic [n = 64], Mississippi [n = 125], Central [n = 143], Pacific [n = 123]) were assigned to a GIS based model [16] of stable isotopes in precipitation (δD_p_) and recalibrating the surface to reflect δD in feathers via regression (see Methods). Assignments were based on a Bayesian framework incorporating a spatially explicit isotope-based likelihood function with spatially explicit prior probabilities of being shot in a given flyway based on banding records (see Methods and Table 2). The posterior probability density for each individual was then converted to a binary surface by assigning a “1” to all grid points with odds greater than 2∶1 relative to the highest density value and a “0” to all others. Each grid point shown here represents the sum of these individual-level binary grids, summed over all individuals.

**Figure 5:**
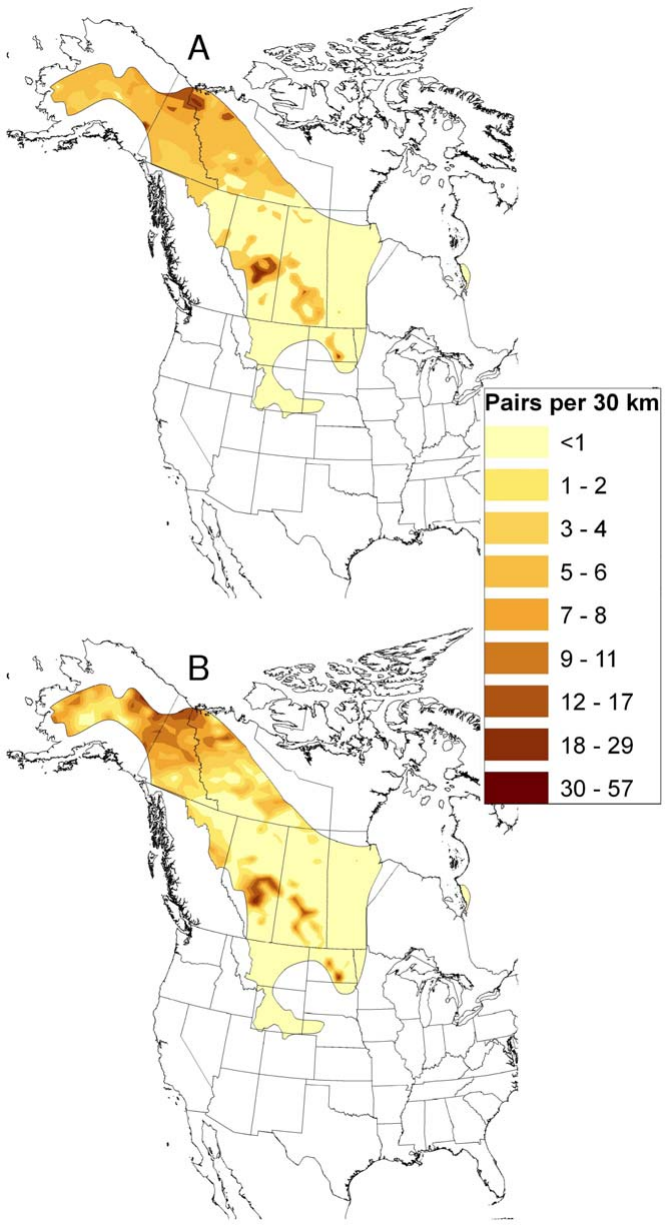
Expected geographic distribution of origins of hatching-year scaup harvested in 1999 and 2000. Expected distributions are based on scaup pair count data (31) interpolated using Ordinary Point Cokriging, with detrending (see Methods and Table 3).

Our geospatial natal assignment model suggested that hunter-killed juvenile scaup originated from throughout their entire known breeding range. However, most of the individuals originated from mid-latitudes within that overall range (Fig. 2). Our sample of juvenile scaup wings was relatively evenly distributed among the four flyways from which they were collected, with 21% collected in the Atlantic flyway, 26% from the Mississippi flyway, 26% from the Central flyway, and the remaining 27% from the Pacific flyway. This contrasts with estimated harvest proportions for the hunting seasons starting in 1999 and 2000 [27]; those estimates suggest that 13% of juvenile scaup were harvested in the Atlantic flyway, 49% in the Mississippi flyway, 24% in the Central flyway, and the remaining 13% in the Pacific flyway. Due to this disparity, we summarized the origin assignments by harvest flyway; these analyses suggest that most of the scaup harvested in the Atlantic, Mississippi, and Central flyways originated from mid latitudes of the boreal forest and Prairie-Parkland regions of Alberta, Saskatchewan and Manitoba (Figs. 3 and 4). The natal assignments suggest that in both 1999 and 2000, juvenile scaup originated from further south than would be expected based solely on breeding pair abundance (Figs. 3 and 4 vs. Figs. 5a and 5b, respectively). Interpolated breeding population data suggest that the vast majority of scaup were encountered north of 60° N latitude in 1999 and 2000, with scattered concentrations in the parkland regions of Alberta, Saskatchewan and adjacent North Dakota (Figures 5a and 5b). As a result, 1999 pair counts were negatively correlated with assigned natal origins of hunter killed (1999 season) scaup (rho = −0.343, p<0.001). Similarly, pair counts in the year 2000 were also negatively correlated with natal origins of scaup in the 2000 hunting season (rho = −0.280, p<0.001)

Juvenile scaup sampled in the Pacific Flyway in 1999 and 2000 were largely assigned to natal origins in central and eastern Alaska (Figs. 3 and 4). Within breeding sites in the Pacific Flyway, scaup breeding population densities showed less structure than east of the continental divide; however, scaup abundance tended to be greatest in eastern Alaska and Yukon (Figs. 5a and 5b). Thus, assigned natal origins (Figs. 3 and 4) of juvenile scaup shot in the Pacific Flyway appeared to be west of the areas with the greatest concentrations of breeding birds in both 1999 and 2000 (Figs. 3 and 4 vs. Figs. 5a and 5b, respectively).

**Figure 6:**
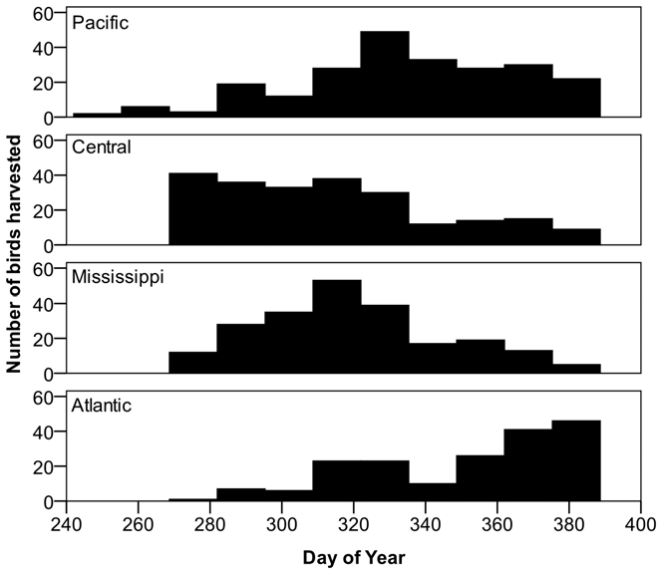
Temporal distribution of scaup harvest in flyways across North America in 1999–2000 and 2000–2001. Note that values for Day of Year extend past 365 due to the addition of 365 to dates in early January to ease interpretation of the temporal distribution.

**Figure 7:**
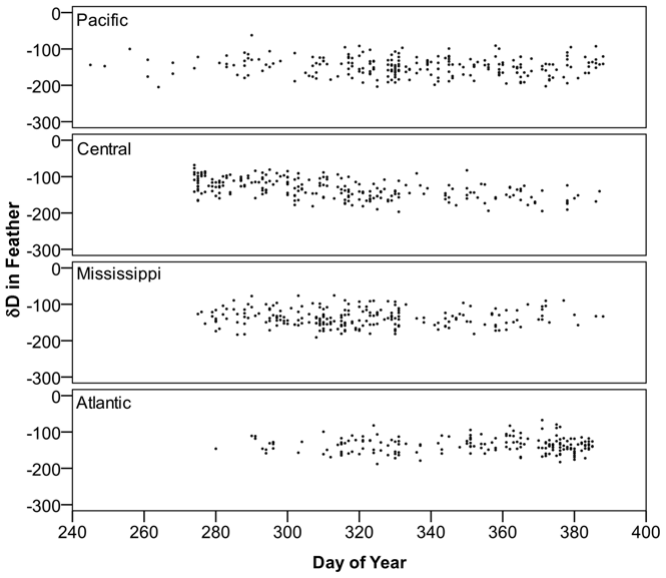
Temporal trend in δD for wing feathers of hatching year scaup harvested across North America. Note that values for Day of Year extend past 365 due to the addition of 365 to dates in early January to ease interpretation of the temporal distribution.

It was possible that time of harvest could have an influence on the distribution of origins for juvenile scaup. For example, if birds from southern natal areas were available to hunters earlier in the season, then early harvest samples would tend to be biased toward birds from southerly latitudes. Although the distributions of harvest times were not identical for samples from each of the four flyways (Fig. 6), there were no strong trends in δD_f_ with time of harvest (Fig. 7), suggesting that time of harvest did not explain the differences among the distributions of geographic origins of scaup harvested in each of the flyways.

## Discussion

By combining observations, stable isotope, and advanced geostatistical approaches to delineating origins of hatch-year lesser scaup we have demonstrated that natal origins of juvenile scaup harvested in 1999–2000 and 2000–2001 were not distributed across the scaup breeding range in direct proportion to estimated abundance of the breeding population. Rather than being harvested in proportion to the species' breeding population abundance, more juveniles were harvested from the central and southern regions of the boreal forest and the Prairie-Parkland than expected, with fewer juveniles from the northwestern boreal forest. This pattern was consistent among years over the two hunting seasons that we examined. For the Pacific population, the disparity was less pronounced due to the resolution of assignments and smaller geographic target area, but again, it appeared as though fewer than expected harvested birds originated from the northern boreal forest. Together, these results suggest that either (1) the south-central boreal and Prairie-Parkland populations were more productive than expected based on breeding population abundance, (2) that the northernmost Boreal region was less productive than expected, or (3) that there was differential susceptibility to hunting depending on the natal origin of individuals. Another potential explanation for this pattern would be that scaup populations in the north had exceeded carrying capacity and that density-dependent mechanisms are responsible for the relatively reduced productivity in the north. This last scenario seems least likely since the overall population size of scaup has been in decline for some time and it is not likely that they have reached carrying capacity in any part of their range. The former three scenarios, however, have important ramifications with respect to likely causes of continental population declines in this species.

In a review of the pattern of population decline in scaup (including both lesser and greater (*A.marila*) scaup) for the period 1955–1997, Boreal populations have shown a steady decline especially over 20 years (1978–1997) whereas Prairie-Parkland populations have fluctuated in relation to the total abundance of May pond habitat [9]. Our analyses suggested that recruitment of lesser scaup into the fall flyway populations differed according to latitudinal zone in the Boreal, with the northern Boreal showing the lowest recruitment relative to breeding population abundance.

Scaup at the northern limits of their range must migrate farther and have shorter overall breeding seasons than those nesting further south. These constraints may make these birds more susceptible to mechanisms of population regulation associated with female body condition, timing of breeding or quality of fledging juveniles. The Spring Condition Hypothesis (SCH) states that declines in female condition may reduce fecundity or breeding success [10], but some evidence is inconsistent with this idea [11]. Very little data are available on scaup duckling survival and recruitment rates in the remote Boreal forest or within the Boreal biome. Based on recent work, lesser scaup duckling survival is reportedly low, but highly variable in Boreal forest of Alaska [28]–[30]. Assuming that lower juvenile recruitment occurs in northern scaup populations, further work is required to evaluate the controlling mechanisms underlying this pattern.

The northern Boreal regions, particularly those areas including wetlands associated with peatlands and permafrost, are susceptible to short- and long-term changes in climate that can impact the local water budget [31]. It is possible that scaup breeding at the northern portion of their range have simply experienced periodic changes in water condition and foodweb productivity, although few data exist to confirm this hypothesis [32]–[35]. Scaup adults and juveniles feed heavily on freshwater amphipods (e.g., *Gammarus lacustris*) and other benthic invertebrates. Both short- and long-term climatically driven hydrologic variability that influence regional changes in the availability of these foods may profoundly influence scaup productivity and recruitment. Other causes of scaup breeding failure include predation on nests, young and adults. Cycles in the abundance of small mammal populations in the Boreal can indirectly influence overall duck productivity by modifying intensity of nest predation [36].

We suggest that demographic studies contrasting lesser scaup productivity in the northern region with central and southern Boreal areas will greatly aid our understanding of continent-wide population declines in this and other wetland-associated species [37], [38]. Because the southern and central Boreal and Prairie-Parkland populations may be providing a disproportionately large component of the hatching-year lesser scaup harvest, future research into this question and the timing of gamebird harvest management relative to origin by flyway is now appropriate.

Determining the relative importance of hypotheses proposed for low or declining scaup populations will be possible when key assumptions and biases have been evaluated. For instance, how effectively the May Waterfowl Breeding Pair surveys provide breeding distribution abundance for lesser scaup is open to debate. However, if anything, the timing of this survey will underestimate the number of breeding pairs at more northerly latitudes [39] and so render our conclusions more conservative. Similarly, it is currently unclear how well the wing surveys represent total hunter harvest. However, our effort was based on the best data available and any future refinements in these techniques or improved estimates of inherent biases can readily be incorporated into our statistical approach.

Our current population models, based on limited data for Boreal-breeding birds, suggest that scaup population dynamics are highly responsive to changes in adult female non-breeding (and breeding) season survival and to a lesser extent on variation in nest and duckling survival rates [40]. If low productivity is more prevalent now than it was historically, it will be important to know whether proposed hunting restrictions for scaup will increase adult and juvenile survival rates to levels that offset this lower production. To inform harvest management, we strongly encourage improved survival analyses for scaup, especially those targeting northern scaup populations.

Finally, we stress here the importance of our new approach to investigating population declines in a broadly distributed migratory species. We chose lesser scaup as an example because of the well documented continent-wide temporal decline in this species, the comparatively extensive surveys conducted to establish the relative abundance of breeding birds across its range, and availability of band recovery information obtained regularly from hunters. Such background data and opportunities are not likely to be available for the vast majority of migratory species in North America.

Our example relies on calibrating an isoscape generated from long-term averaged isotope data for precipitation as the explanatory spatial process. Our application therefore assumes that patterns in δD_f_ across geography are very general and stable signals derived from averages in δD_p_. In our case, the calibration explained about 64% of the variation in feather data, which leaves just less than half of the variance unexplained. It may be possible that spatially based variance generating processes can be better modeled without using the intermediate precipitation based isoscape as illustrated here; a more spatially exhaustive sampling frame for feathers would be required to make such a comparison. We encourage such comparisons for cases where highly structured and exhaustive spatial sampling can be carried out within a narrow time window (e.g. over one or two years). More generally, as breeding and wintering ground surveys improve for other migratory birds, including non-game species, we encourage others to not only test methodological assumptions and hypotheses, but also to continue rigorously evaluating hypotheses about factors affecting population dynamics and distributions in light of novel methodological approaches.
